# Tuning the Physical State of Aripiprazole by Mesoporous Silica

**DOI:** 10.1021/acs.molpharmaceut.3c01095

**Published:** 2024-04-22

**Authors:** Daniel Kramarczyk, Justyna Knapik-Kowalczuk, Joanna Klimontko, Mateusz Kurek, Renata Jachowicz, Marian Paluch

**Affiliations:** † Faculty of Science and Technology, Institute of Physics, University of Silesia in Katowice, SMCEBI, 75 Pułku Piechoty 1a, 41-500 Chorzów, Poland; ‡ Department of Pharmaceutical Technology and Biopharmaceutics, Faculty of Pharmacy, Jagiellonian University, Medyczna 9, 30-688 Kraków, Poland

**Keywords:** aripiprazole, amorphous, physical stability, polymorphism, silica materials

## Abstract

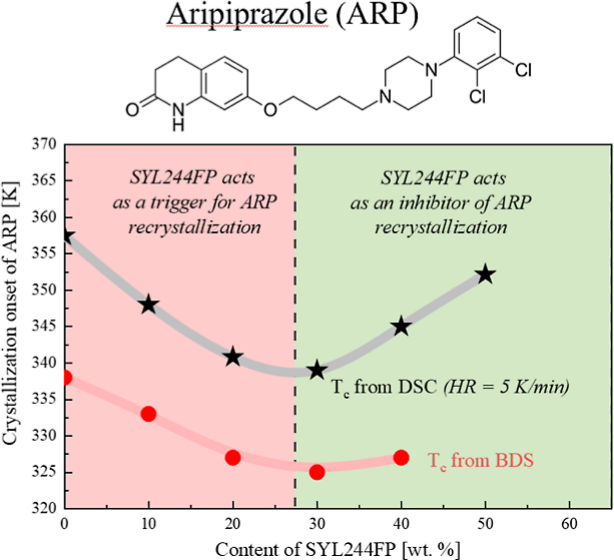

The main purpose of our studies is to demonstrate that commercially available mesoporous silica (MS) can be used to control the physical state of aripiprazole (ARP). The investigations performed utilizing differential scanning calorimetry and broadband dielectric spectroscopy reveal that silica can play different roles depending on its concentration in the system with amorphous ARP. At low MS content, it activates recrystallization of the active pharmaceutical ingredient and supports forming the III polymorphic form of ARP. At intermediate MS content (between ca. 27 and 65 wt %), MS works as a recrystallization inhibitor of ARP. At these concentrations, the formation of III polymorphic form is no longer favorable; therefore, it is possible to use this additive to obtain ARP in either IV or X polymorphic form. At the same time, employing MS in concentrations >65 wt % amorphous form of ARP with high physical stability can be obtained. Finally, regardless of the polymorphic form it crystallizes into, each composite is characterized by the same temperature dependence of relaxation times in the supercooled and glassy states.

## 1 Introduction

The manipulation of the physical state of active pharmaceutical ingredients (APIs) is an important and innovative field of pharmaceutical science. This is because by altering the physical form of API, one can improve, among others, the dissolution properties of drug compounds, ultimately increasing their bioavailability and therapeutic efficacy.^1−4^ It is crucial, especially because approximately 40% of marketed drugs reveal poor aqueous solubility.^5−8^ Furthermore, it is anticipated that up to 90% of new chemical entities will encounter this issue and thus might be rejected from the research and development pipeline.^5,9^ The recalled statistics highlight the significance of developing effective strategies to improve the solubility of drugs and thus enhance their therapeutic efficacy and patient outcomes. In the literature, many reports prove that API’s aqueous solubility, bioavailability, and dissolution rate can be improved even a dozen times when it is converted to an amorphous or metastable polymorphic form.^10−15^

In general, the improved solubility of pharmaceuticals after conversion to metastable polymorph is associated with differences in molecular packing and surface area in their crystal structure.^12,16^ For example, the packing of molecules in the metastable form may be less dense, which can increase the free energy of the system and enhance the solubility. Unlike any polymorphic form of crystalline material, amorphous material does not have a regular arrangement of atoms in a repeating pattern. The lack of long-range molecular order and associated high Gibbs free energy are reasons for its unique properties, including increased solubility and bioavailability compared with their crystalline counterparts. In both described approaches (i.e., a conversion of API having solubility-limited bioavailability to its metastable polymorph or amorphous form), the advantages come at the cost of the material’s physical stability. Due to its disordered nature associated with that highest internal energy, an amorphous form reveals the most prominent tendency toward recrystallization.^17−21^ In comparison, the metastable polymorph reveals better physical stability than its amorphous counterparts but might also possess a lower solubility. Usually, developing a selective, fully controlled, and reproducible technology for obtaining a specific metastable polymorph of a drug is not easy. It requires a lot of work and time and, frequently, it turns out that the conditions needed for its formation may be difficult to repeat, resulting in problems in manufacturing procedures.^22,23^

In this paper, the impact of mesoporous silica (MS) on the physical state of aripiprazole (ARP) will be presented. The use of ARP, an atypical antipsychotic employed in treating various mood and psychotic disorders, as a model drug was essential. On the one hand, according to the classification system introduced in 2010 by Baird et al.,^24,25^ ARP belongs to the second group of glass-forming substances. This classification divides organic molecules into three classes and links the glass-forming ability of the material with its crystallization tendency from the melt when treated in a particular way (N_2_ atmosphere; heating rate 10 K/min; cooling rate 20 K/min; reheating rate 10 K/min). The first class includes nonglass-formers, i.e., compounds that crystallize on cooling the melt at a temperature lower than the melting temperature. Glass formers, which crystallize on heating the melt-quenched material (above its glass transition temperature (*T*_g_)), such as ARP, were defined as class two compounds. At the same time, the third class contains compounds that show no sign of crystallization on heating after the melt-quenching. Consequently, using ARP as a model system, it is possible to investigate, in a relatively quick time, whether the employed MS will effectively improve the physical stability of the amorphous form of ARP. On the other hand, this particular API is characterized by unique structural flexibility, allowing different structural conformations and resulting in nine different polymorphs.^26−29^ This makes ARP one of the most polymorphically rich organic crystals discovered so far. Therefore, it will be interesting to check how the employed MS affects the recrystallization tendency of ARP and which polymorphic form is preferred in the ARP–MS composite. The effect of the MS concentration on the formation of different ARP polymorphic forms will also be examined. Consequently, our experiments will assess the mechanism of ARP’s stabilization by the MS.^30−36^

To solve all the above issues, this paper uses the neat APR and systems containing ARP and 10, 20, 30, 40, and 50 wt % of Syloid 244FP (SYL244FP). All composites were thoroughly investigated by differential scanning chromatography (DSC) and broadband dielectric spectroscopy (BDS). The results obtained indicated that the chosen MS at low concentrations works as a trigger for ARP’s recrystallization, but after reaching a certain content, it starts to play a role as a stabilizer. We show that the observed changes in the stabilization of the ARP amorphous form are associated with the inhibition of nucleation of its III polymorphic form. Consequently, our studies have shown that employing an appropriate amount of MS can control the ARP’s physical state.

## 2 Materials and Methods

### 2.1 Materials

ARP with purity ≥99.0% and molecular mass *M*_w_ = 448,4 g/mol was purchased from HyperChem (Zhejiang, China); ARP is chemically described as 7-[4-[4-(2,3-dichlorophenyl)piperazin-1-yl]butoxy]-3,4-dihydro-1*H*-quinolin-2-one. Syloid 244FP (SYL244FP) was received as a gift from Grace GmbH & CO. KG (Worms, Germany). This MS is characterized by an average particle size of 2.5–3.7 μm, a surface area of 314 m^2^/g, a pore diameter ∼23 nm, and the pore volume equal to 1.6 mL/g. All chemicals were used as received.

### 2.2 Sample Preparation

Binary mixtures containing ARP and 10, 20, 30, 40, 50, and 65 wt % of SYL244FP, respectively, were prepared by physically mixing in a mortar. The procedure consisted of about 3 min of mixing, and then the sample was scraped off the mortar wall with a spatula. The mixing procedure was repeated three times. Before each experiment, pure ARP and systems were dried at 373 K for 10 min to remove water contribution. In the BDS experiments, the samples were placed in a parallel-plate cell made of stainless steel (diameter 15 mm and a 0.1 mm gap provided by silica spacer fibers) and then melted in the hot plate at 421 K and quenched on a copper plate. The melting procedure occurred under air conditions with an environmental humidity of approximately 25% RH. In the DSC experiments, the samples were placed in aluminum crucibles (40 μL) and vitrified in situ in the apparatus under dry nitrogen conditions.

### 2.3 Differential Scanning Calorimetry

Thermal properties of pure ARP and its mixtures with SYL244FP were investigated using a Mettler-Toledo DSC 1 STARe System. The DSC was calibrated for temperature and enthalpy using zinc and indium standards. The instrument had an HSS8 ceramic sensor with 120 thermocouples and a liquid nitrogen cooling station. The measurements were carried out with a heating rate of 10 or 5 K/min. The obtained DSC thermograms were analyzed in Origin (OriginLab Corporation, Northampton, MA, USA) using Multiple Peak Fit analysis based on the Gaussian model. The available tools allowed for a detailed analysis of the melting processes, which was shown in the DSC analysis of pure ARP.

### 2.4 Broadband Dielectric Spectroscopy

The dielectric measurements of pure ARP and its mixtures containing 10, 20, 30, 40, and 50 wt % of SYL244FP were performed using a Novo-Control GMBH Alpha dielectric spectrometer (Montabaur, Germany). The temperature in this apparatus was controlled by a Quattro temperature controller with temperature stability better than 0.1 K. Nonisothermal studies of ARP + 10, 20, 30, 40, and 50 wt % of SYL244FP were performed in the temperature range from 153 to 308 K with a step of 5 K and from 310 to 342 K with a step of 2 K in a broad frequency range from 10^–1^ to 10^6^ Hz.

### 2.5 X-ray Diffraction

The X-ray diffraction (XRD) studies of powdered samples (ARP + 10% SYL244FP, ARP + 30% SYL244FP, and ARP + 50% SYL244FP) were performed with a Malvern Panalytical Empyrean diffractometer (Malvern Panalytical Ltd., Malvern, UK) using a nickel filtered Cu Kα_1,2_ source (λ = 1.5406 Å) and equipped with a PIXcell^3D^ ultrafast solid-state hybrid detector. Measurements were carried out at room T condition, in reflection mode in the Bragg–Brentano geometry, within the scattering angle 2θ range of 5–70°. Prior to the XRD experiment, the samples (i.e., physical mixtures) were quench cooled, then annealed at 313 K for 20 h, and recrystallized at 353 K for 48 h.

## 3 Results and Discussion

### 3.1 Thermal Properties of Neat ARP

**Figure 1:**
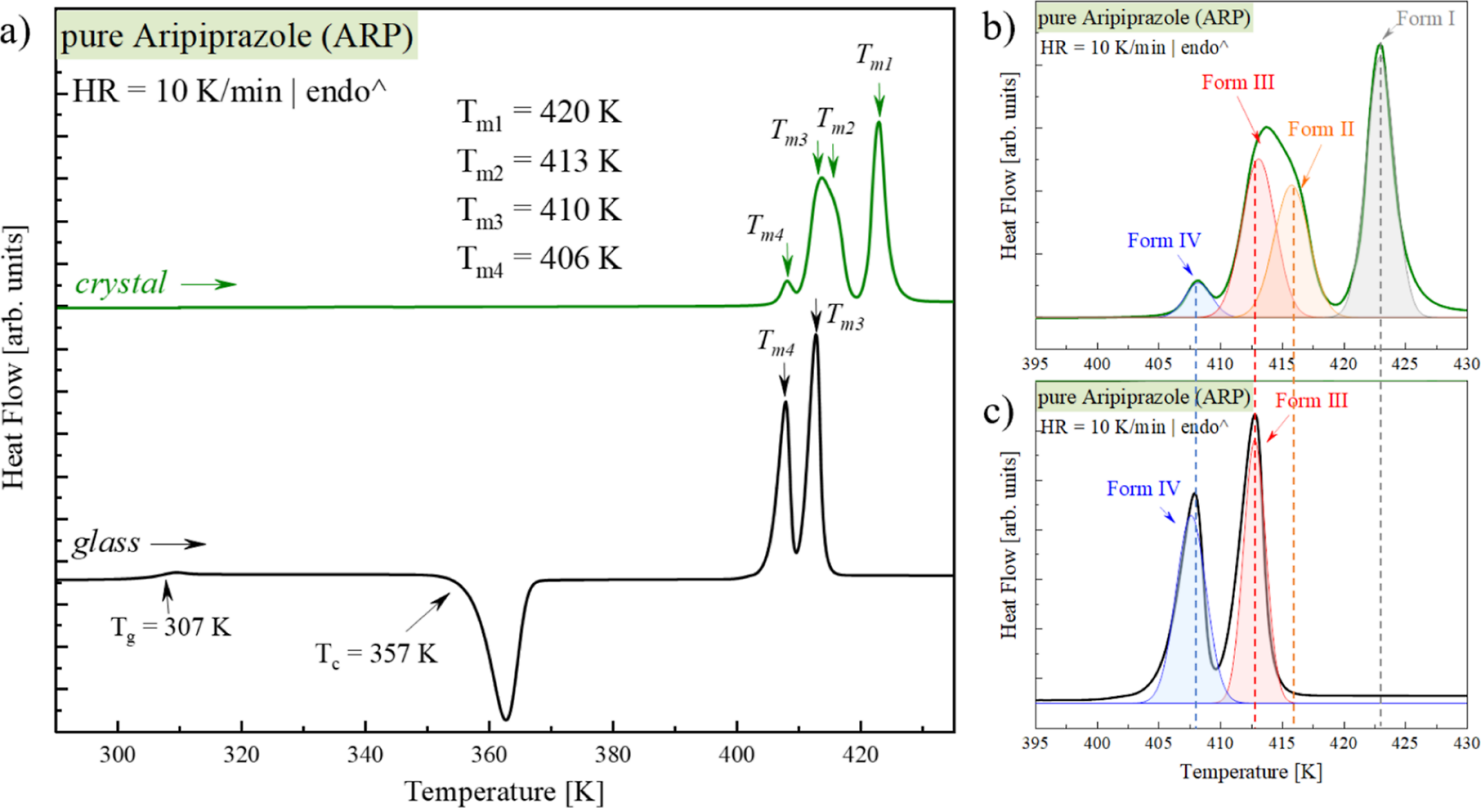
In panel (a), DSC thermograms of crystalline and amorphous ARP are presented. Panels (b,c) present the zoomed areas of meltings.

The thermal properties of pure ARP were investigated using DSC. First, crystalline ARP was heated from 280 to 433 K with a heating rate (HR) of 10 K/min. Further, the sample was vitrified in DSC and subsequently reheated with the same temperature range and HR. As presented in Figure 1, the crystalline ARP is characterized by four endothermic peaks, which indicate four different polymorphic forms in the examined sample. These processes are labeled from the right to the left. Consequently, the process with the highest melting temperature has been called form I, and the others have been consistently labeled as form II, III, and IV. The melting points were determined at the onset of the process at temperatures equal to *T*_m I_ = 421 K, *T*_m II_ = 414 K, *T*_m III_ = 410 K, and *T*_m IV_ = 406 K. It is worth noting that peaks characterizing form II and form III overlap, therefore to determine their onsets and peak temperatures, the Multiple Peak Fit analysis in the Origin software was performed. The results are summarized in Table 1, supplemented by the literature data.

**Table 1:** Comparison of the Melting Point Values at the Place of the Beginning and the Maximum for the Obtained Forms During DSC Measurements^a^

form	onset temperature (K)		peak temperature (K)	
I	420.5	422.3^b^	423.0	422.7^c^
II	414.4	416.3^b^	416.5	417.8^c^
III	410.3	412.4^b^	413.8	412.7^c^
IV	406.3	408.1^b^	408.1	408.9^c^

a Table also includes literature values.

b Values taken from Braun et al. (2009).^26^

c Data digitalized from Braun et al. (2009).^26^

Returning to Figure 1, a step-like behavior was revealed during the heating of the amorphous form of ARP, reflecting the glass transition at *T*_g_ = 307 K. Further heating of the sample showed a single exothermic process corresponding to a recrystallization that begins at *T*_c_ = 357 K, followed by the three endothermic processes reflecting sample melting. As can be seen, the initial crystal of ARP has a different melting characteristic in comparison to the sample after vitrification and recrystallization. The ARP’s crystal obtained from devitrification does not form I and II polymorphic forms (which are dominant in the starting material). Instead, it combines III and IV polymorphs.

### 3.2 Molecular Dynamics of Neat Amorphous ARP

As mentioned in the previous section, the main limitation of using amorphous APIs in the pharmaceutical industry is their poor physical stability. Many factors may cause disordered materials to recrystallize; however, the material’s molecular mobility is often considered crucial.^37−45^

**Figure 2:**
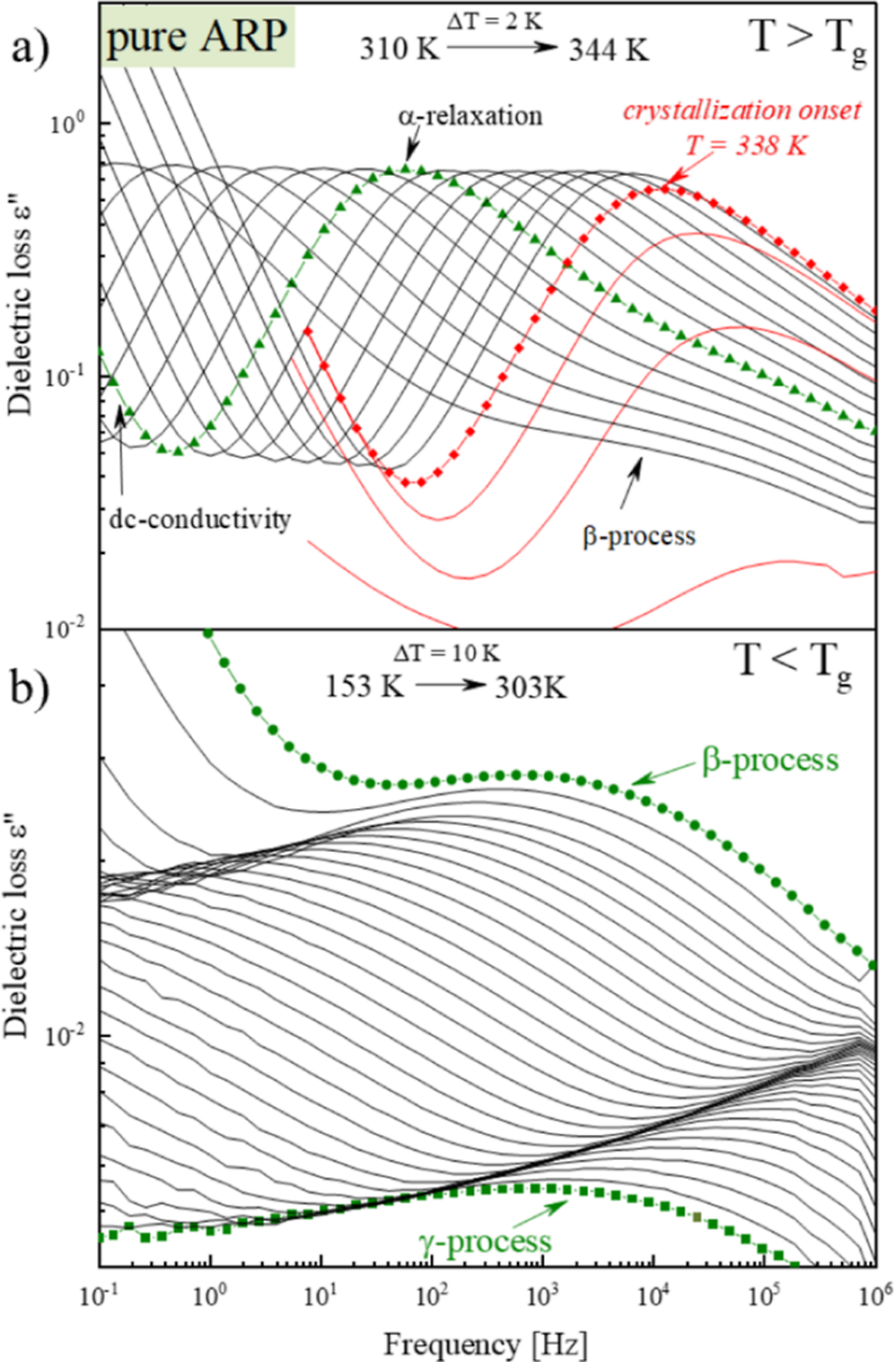
Dielectric spectra of amorphous ARP obtained (a) above *T*_g_ and (b) below *T*_g_.

The dielectric loss spectra were measured using the BDS technique to investigate the molecular dynamics of neat ARP in both glassy and supercooled liquid states. In this experiment, the temperature was increased from 153 to 308 K in the 5 K step (i.e., in the glassy state) and from 310 to 342 K in the 2 K step (i.e., in the supercooled liquid state). The representative spectra are shown in Figure 2. For better visualization, the data are divided into two panels presenting spectra collected at temperatures above and below the glass transition temperature of APR. As can be seen, three main features characterize the spectra of ARP collected at *T* > *T*_g_ (panel a). On the low-frequency side, the DC conductivity associated with the translational motion of residual ion impurities can be distinguished. Next, looking toward higher frequencies, two relaxation processes are visible: (i) very well-pronounced structural (α) relaxation, which reflects the cooperative motions of entire molecules, and (ii) the barely seen secondary (β) process. As can be seen, the α-relaxation peak is shifted toward higher frequencies with increasing temperature. Note that at 338 K, a drastic drop in the α-relaxation peak intensity was registered. Such behavior is a manifestation of sample recrystallization. This is a consequence of the reduction in the total number (N) of actively reorienting dipoles (μ), which contribute to the structural relaxation process once the fraction of the amorphous phase decreases ().^46^

Since the secondary relaxation processes originate from the local (intra- or intermolecular) motions of the molecules, they are much faster than the structural relaxation. These relaxation processes are usually better visible on the dielectric loss spectra collected at temperatures below the material’s *T*_g_ (i.e., in a glassy state). As shown in Figure 2b, ARP is characterized by two secondary relaxation processes. The nature of both of these secondary relaxations will be discussed later in this paper.

**Figure 3:**
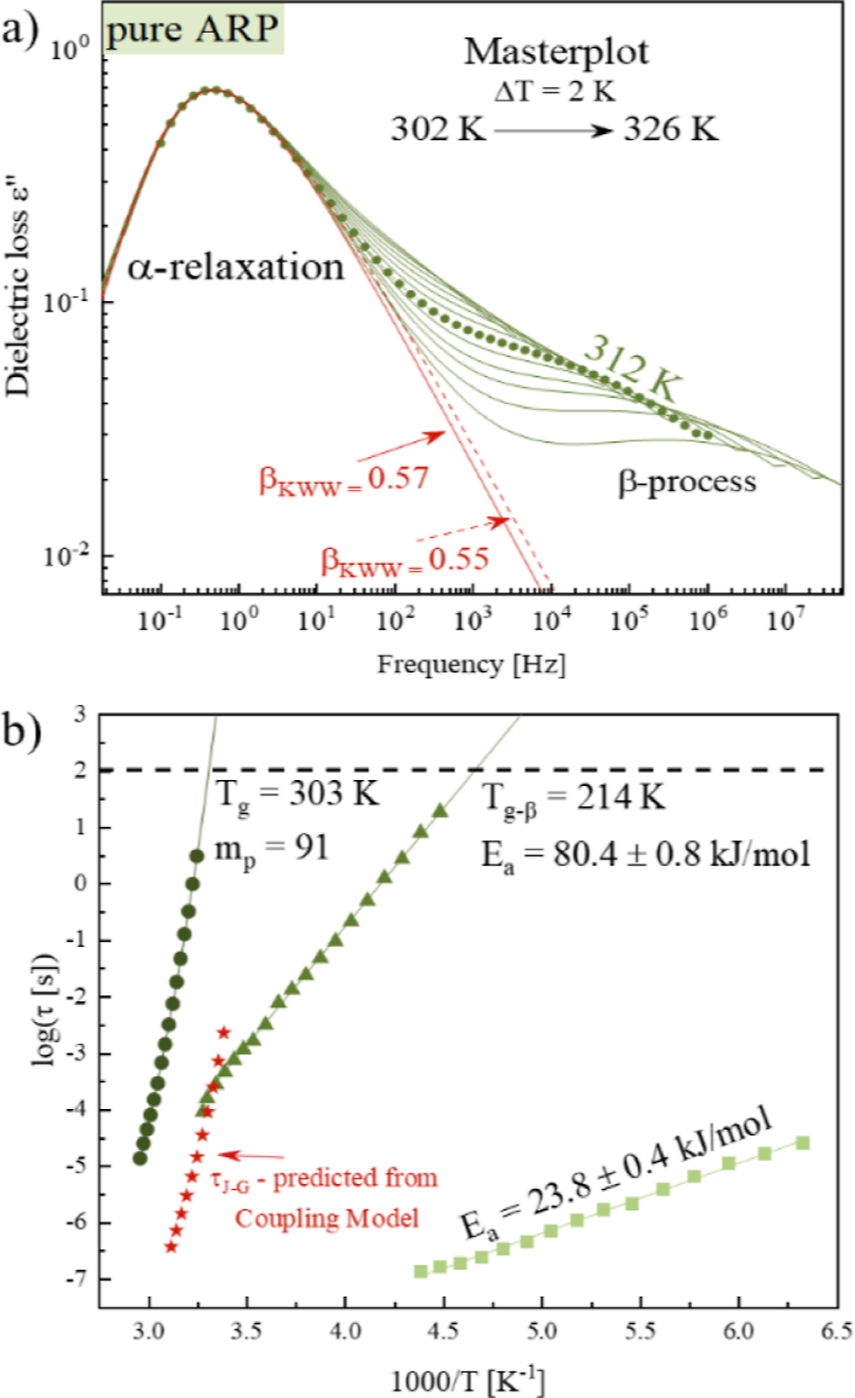
Panel (a) presents the analysis of the widening of the structural relaxation process and (b) presents the analysis of relaxation times of ARP.

To check whether or not the shape of the α-relaxation peak remains constant in the whole examined temperature range, a so-called masterplot has been constructed (see Figure 3a) by the horizontal shifting of dielectric spectra taken at temperatures from 302 to 326 K to superimpose on the reference spectrum at 312 K. Next, the Kohlrausch–Williams–Watts (KWW) function^47^ has been used to describe the shape of dielectric loss spectra (see the red lines in Figure 3a). The stretch exponent in the KWW function, β_KWW_, takes values from 0 to 1. The value of 1 represents the narrow and symmetrical peak. When the β_KWW_ value decreases, the α-peak widens and becomes increasingly asymmetric. In the case of ARP, an increase in the temperature causes a slight broadening of the α-peak. For the lowest presented temperature, the β_KWW_ parameter is 0.57, while for the temperature higher by 24 K, β_KWW_ is 0.55. In this case, the α-peak broadening phenomenon is due to the presence of a secondary β-process on the right-side wing of the α-process that widens the structural relaxation peak.

From further analysis of ARP’s dielectric loss spectra, the temperature dependences of the relaxation times of the α-, β-, and γ-relaxation processes were obtained (see Figure 3b). For this purpose, the asymmetric structural relaxation process as well as symmetric secondary relaxation processes have been fitted using the Havriliak–Negami (HN) and the Cole–Cole (CC) functions, respectively. The empirical HN is defined as follows^48^
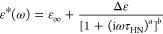
1where ε_∞_ is the high-frequency limit permittivity, ε_0_ denotes the permittivity of vacuum, Δε is dielectric strength, ω is equal to 2π*f*, τ_HN_ is the HN relaxation time, and *a* and *b* represent symmetric and asymmetric broadening of the relaxation peak. When the *b* parameter is equal to 1, the HN function becomes the CC function, which was used to fit the secondary relaxations of ARP. The obtained fit parameters were then used to calculate the τ_α_, τ_β_, and τ_γ_ in accordance with the equation
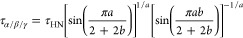
2

As can be seen in Figure 3b, in the supercooled liquid region, the temperature evolution of the structural (α) relaxation time of ARP (represented as a circle points) can be well described by the Vogel–Fulcher–Tammann (VFT) equation^49−51^
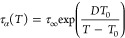
3with corresponding fitting parameters equal to log_10_(τ_∞_) = 15.73 ± 0.78, *T*_0_ = 248.0 ± 0.3 K, and *D* = 2257 ± 11. To estimate the kinetic glass transition temperature of the investigated API, the commonly known definition of *T*_g_ = *T*(τ_α_ = 100 s) was employed. The extrapolation of τ_α_(*T*) dependence to τ_α_ = 100 s gives the value of the glass transition temperature equal to *T*_g_ = 303 K. It is worth noting that this value corresponds well with that obtained from DSC experiments. A slight difference in these values results from the differences in the heating rates employed in BDS and DSC experiments. Based on VFT fits, one can also calculate the steepness index (*m*_p_), also called the fragility parameter, which is defined as follows^52^
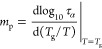
4

The typical values of the steepness index for various materials vary between 16 and 200. The determined fragility parameter of ARP is equal to *m*_p_ = 91.

Now, we return to the discussion on the molecular origin of the ARP’s secondary relaxations. As can be seen in Figure 3b, in the glassy state, the temperature evolutions of both τ_β_ and τ_γ_ exhibit a linear behavior. Thus, these dependencies can be well parametrized by the Arrhenius equation, defined as follows
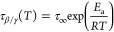
5where τ_∞_ is the pre-exponential factor, *E*_a_ is the energy barrier, and *R* is the gas constant. The resulting fit parameters of ARP’s secondary processes are collected in Table 2.

**Table 2:** Fit Parameters are for Secondary Relaxation Processes^a^

secondary relaxation	log τ_∞_	*E*_a_ [kJ/mol]	type/origin
β	–17.57 ± 0.01	80.4 ± 0.8	JG/intramolecular
γ	–12.39 ± 0.11	23.8 ± 0.4	non-JG/intermolecular

a The table also shows the type and the origin of the process.

Secondary relaxations might be of two types: intra- or intermolecular secondary relaxations. The intramolecular secondary relaxations, also known as non-Johari–Goldstein (non-JG) processes, originate from motions that involve only a subset of the entire molecule. Meanwhile, the intermolecular secondary relaxations, called Johari–Goldstein (JG) processes, come from the local motions of the whole molecule.^53^ Taking into account that the latter are believed to be precursors of structural relaxation, it can be responsible for the recrystallization process of amorphous APIs. Consequently, from the point of view of physical stability, it is important to identify the molecular origin of the secondary relaxations of ARP. For this purpose, we applied the coupling model according to which τ_JG_ is related to the α-relaxation time (τ_α_) as follows
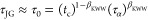
6herein, τ_0_ is the primitive relaxation time, while *t*_c_ is the onset time of intermolecular coupling, which for small molecules (such as ARP) is equal to 2 ps. The values of τ_JG_ determined based on the recalled approach for several temperatures above *T*_g_ of ARP are shown as red stars in Figure 3b. Since the temperature dependence of τ_JG_ appears to be the continuation of the experimentally obtained τ_β_(*T*) one can classify the β-relaxation of ARP as the JG process. At the same time, γ-relaxation is a non-JG processes. The observed complex molecular mobility of amorphous ARP (including the presence of the JG process–the precursor of structural relaxation) well reflects the limited physical stability of this material, which was revealed during both nonisothermal BDS and DSC experiments.

### 3.3 Influence of MS on the Thermal Properties of Supercooled ARP

**Figure 4:**
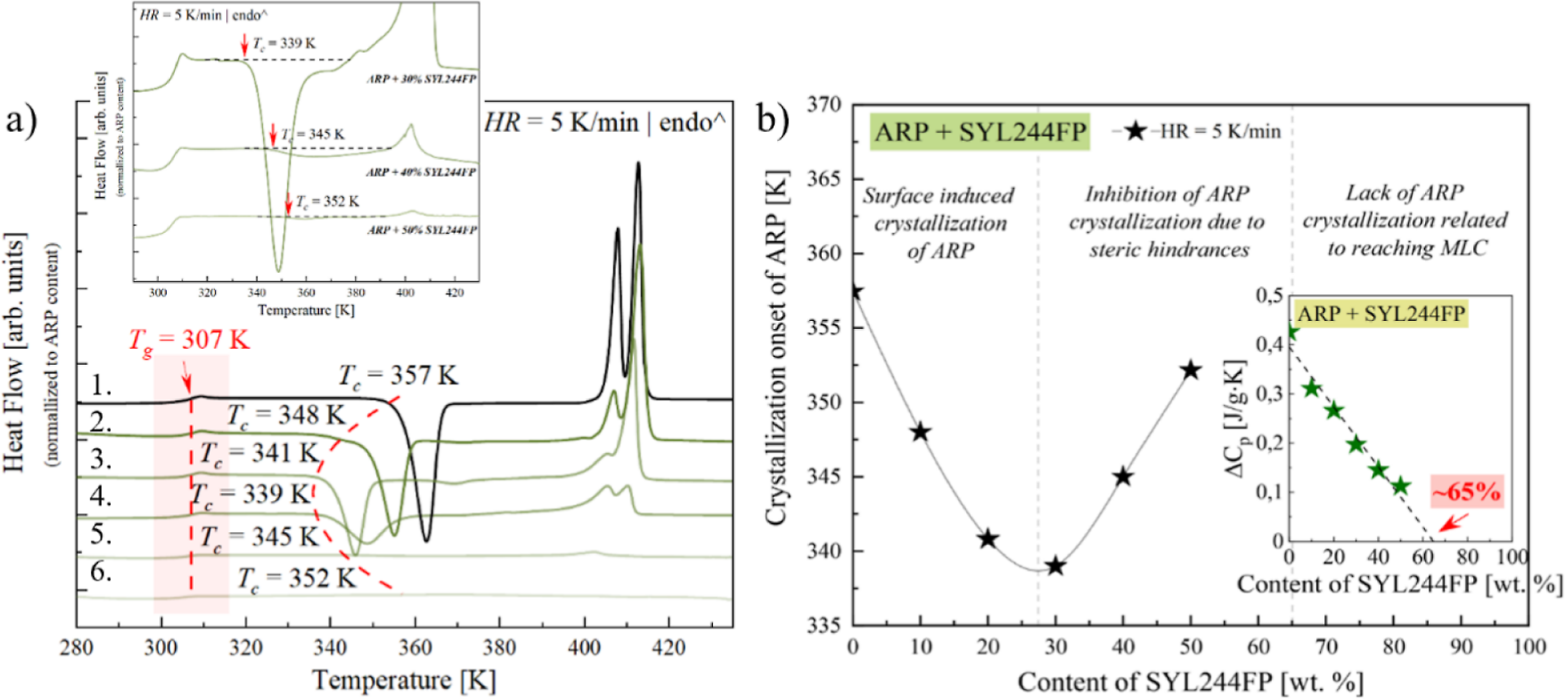
Panel (a) presents DSC thermograms of vitrified ARP and the systems ARP with SYL244FP; inset presents the zoomed area of the panel (a). Panel (b) presents the dependence of the crystallization temperature on the concentration of the tested systems. Inset presents the Δ*C*_p_ dependence on the content of SYL 244FP [wt %].

To investigate the influence of MS on the thermal properties of supercooled ARP, the systems containing ARP and 10, 20, 30, 40, and 50 wt % of SYL244FP have been measured nonisothermally using the DSC. Samples were vitrified in DSC and subsequently reheated from 280 to 433 K with an HR of 5 K/min. Each experiment was performed in triplicate, while the representative DSC traces are presented in Figure 4a.

As can be seen, the employed MS has no impact on the glass transition temperature of ARP. All systems are characterized by the same value of *T*_g_ equal to 307 K. A similar pattern of behavior has been previously found in the cases of simvastatin and celecoxib. However, with increasing SYL244FP, one can observe the decrease in Δ*C*_p_ at the glass transition temperature (*T*_g_). This behavior results from the extensive and additive properties of Δ*C*_p_. The value of Δ*C*_p_ is proportional to the amorphous fraction of API and decreases linearly with decreasing drug content. If drug molecules are absorbed on the surface of MS, they are not contributing to any thermal event since they are “immobilized” through interactions with the functional groups of the MS surface. Consequently, by determining the Δ*C*_p_ value and extrapolation to zero, one can evaluate the monomolecular loading capacity (MLC) of the drug in the MS. The described approach has been introduced by Hempel et al. Based on this method,^54^ the MLC of ARP molecules on the surface of SYL244FP was determined. For that purpose, the Δ*C*_p_ of ARP-SYL244FP was plotted as a function of MS concentration (see the green stars in the inset of Figure 4b). Subsequently, the experimentally determined dependence was parametrized by a linear function. From the fit extrapolation to Δ*C*_p_ = 0, the SYL244FP content, which guarantees enough space to form MLC of ARP on the silica surface, was determined to be 65% (see the dashed line in the inset of Figure 4b). Herein, it is worth pointing out that the composition corresponding to the MLC of the drug molecules on the silica surface is believed to provide a high physical stability of the API. This is connected with the “immobilization” of the drug molecules on the silica surface that finally blocks the drug recrystallization.

To recognize how the employed silica modifies the physical stability of ARP, we analyzed the exothermal processes associated with the recrystallization of ARP from the systems containing different SYL244FP concentrations. As can be seen on the thermograms presented in Figure 4a, for low concentrations of SYL244FP (up to ca. 30 wt %), the additive facilitates ARP’s recrystallization, which is reflected in the significant shift of the onset of the API crystallization process to the lower temperature. After reaching some critical concentration (attributed to ARP + 27.3 wt % SYL244FP), the onset of ARP’s recrystallization shifts toward higher temperatures with increasing SYL244FP content. From that moment, the stabilizing effect dominates until it reaches a concentration that provides the MLC of the drug molecules on the silica surface, for which crystallization should not occur. Thus, for higher silica concentrations (>30 wt %), the excipient becomes the stabilizer of the API. The described behavior, i.e., modification of recrystallization onset of ARP in the presence of the MS, has been graphically shown in Figure 4b.

**Figure 5:**
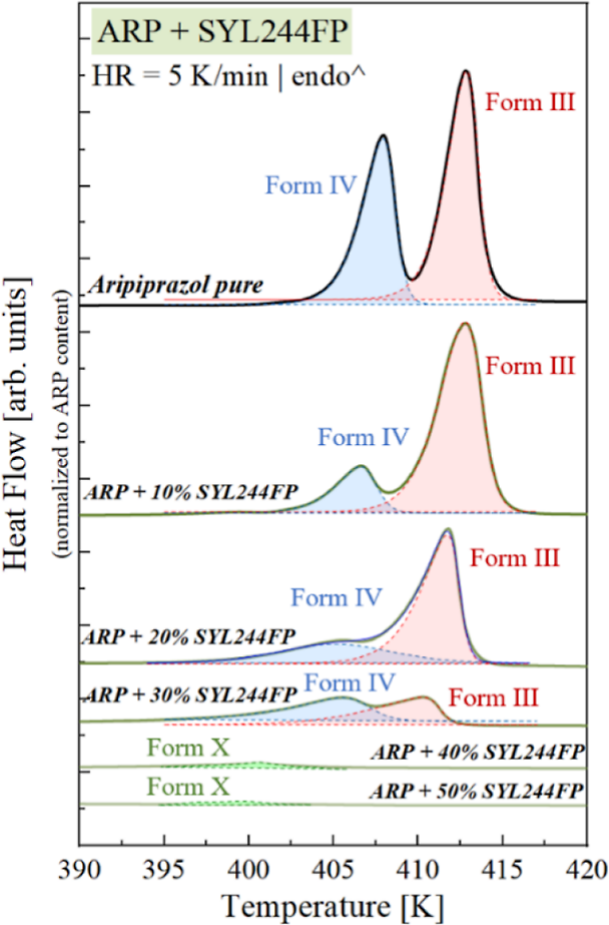
DSC thermograms of vitrified ARP and the systems ARP with SYL244FP that have been normalized to the amount of ARP in the system.

To answer the question of why, at low concentrations, MS triggers ARP’s recrystallization, while at higher concentrations, it works as a stabilizer, it is worth analyzing the melting endotherms of ARP obtained after heating the ARP + SYL244FP systems. For comparative analysis, the obtained heating curves were normalized to the amount of ARP present in a given sample since silica does not contribute to the melting process (Figure 5a). Based on this analysis, one can notice that first (i.e., up to a concentration containing 20 wt % of SYL244FP), form III dominates over the other polymorphic forms of ARP (i.e., form IV). For concentrations equal to 30 wt % of SYL244FP, the contribution of other crystalline fractions (for instance, no IV) becomes more pronounced. Consequently, form III is no longer dominant over the other ARP’s polymorphs. Further increasing the SYL244FP content leads to a substantial decrease in the API’s tendency to recrystallize. Furthermore, during the devitrification, some other polymorph appeared.

Considering all of the above, it has been hypothesized that SYL244FP modifies the recrystallization behavior of ARP by affecting its nucleation. To verify this hypothesis, one should first investigate the impact of the employed MS on the molecular dynamics of ARP. As mentioned in Section 3.2, molecular dynamics is believed to be the critical factor governing the physical stability of amorphous materials. Until now, we have discovered that the employed silica does not plasticize or antiplasticize ARP (has no impact on the API *T*_g_). Further studies have been performed to investigate whether SYL244FP affects the temperature evolution of structural and/or secondary relaxation times, as well as the shape of these processes.

### 3.4 Influence of MS on the Molecular Dynamics of Both Glassy and Supercooled ARP

**Figure 6:**
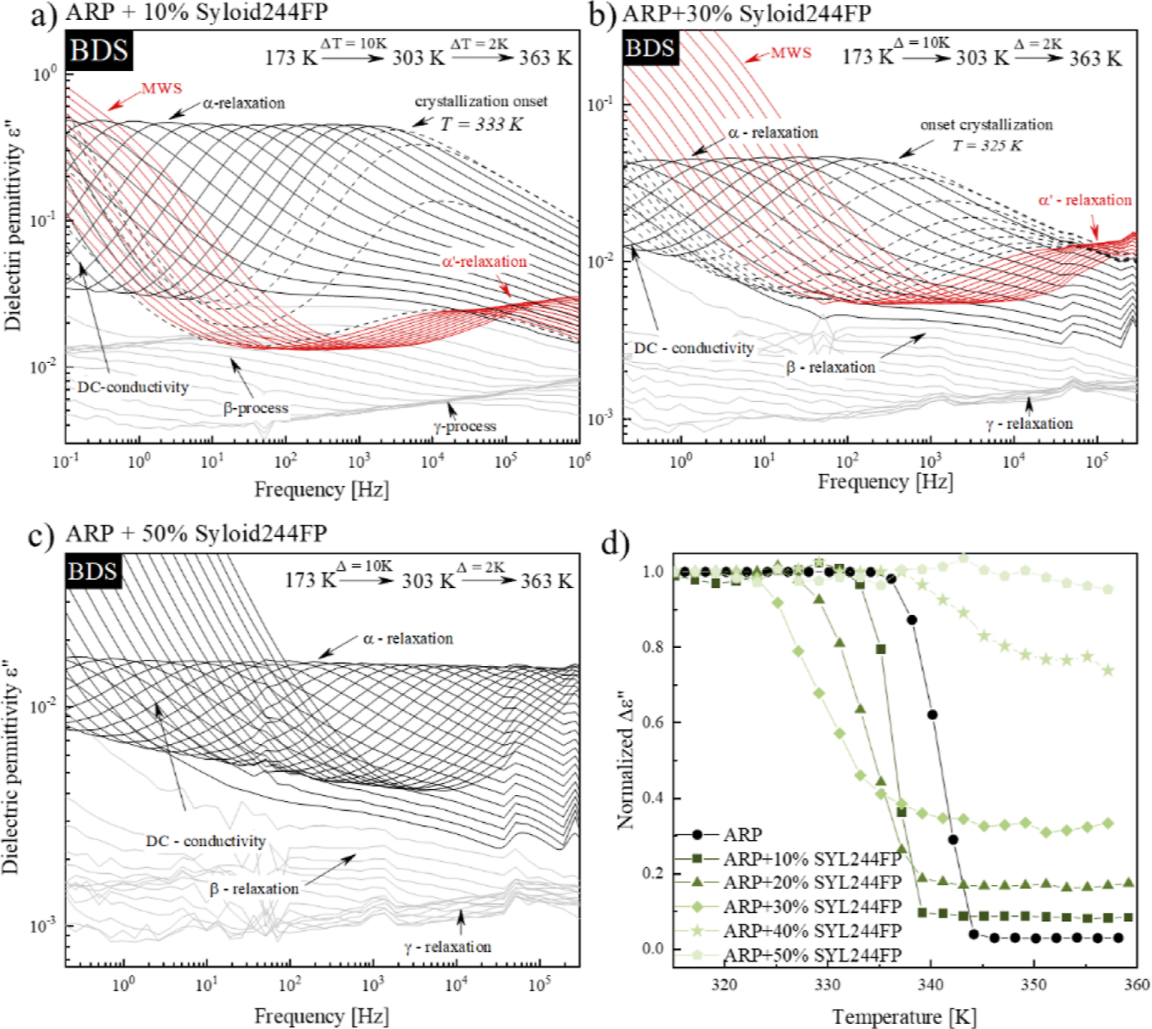
Dielectric loss spectra of ARP + SYL244FP containing (a) 10, (b) 30, and (c) 50% of silica. Panel d presents the normalized value of Δε.

To provide a complete picture of how the employed MS impacts the ARP’s molecular dynamics, the binary systems containing ARP and 10, 20, 30, 40, and 50% of SYL244FP were investigated using the BDS. The dielectric loss spectra were measured on heating at temperatures ranging from 173 to 303 K in step of 10 K, and from 305 to 363 K in step of 2 K. The representative spectra, i.e., for systems containing 10, 30, and 50% of SYL244FP, are shown in Figure 6. Gray lines represent spectra collected at *T* < *T*_g_, and black at *T* > *T*_g_, dashed black lines indicate spectra measured during the recrystallization process, while spectra marked as red lines were measured after the recrystallization. With increasing SYL244FP content, a decrease in the dielectric response is noted, which is obviously connected with the reduction of the ARP fraction in the system (silica does not contribute to the dielectric response). Regardless of the amount of silica used in the composite, the dielectric loss spectra of ARP recorded at *T* < *T*_g_ are characterized by two secondary relaxation processes—β and γ, whose peaks shift toward higher frequencies with increasing temperature. On the other hand, at *T* > *T*_g_, three main features can be noted on the ARP’s spectra. On the low-frequency side, the DC conductivity can be distinguished. Next, the very well-pronounced α—relaxation and secondary β—process are visible.

It should be noted that for concentrations from 10 to 40% of SYL244FP, the recrystallization of ARP from the system was observed as a drastic drop in the intensity of the α-relaxation peak. The onset of recrystallization was registered at 333, 327, 325, and 337 K for systems containing 10, 20, 30, and 40% SYL244FP, respectively. Consequently, the dielectric and calorimetric studies reveal similar recrystallization behaviors of ARP in MS composites. For a low silica content, the additive accelerates API recrystallization. However, after reaching a concentration of 30%, the silica material suppressed ARP’s recrystallization and became its stabilizer. For example, there is no recrystallization during the nonisothermal dielectric measurement of the sample containing 50% of SYL244FP (see Figure 6c). At this point, it is worth noting that ARP’s recrystallization is always incomplete in the SYL244FP composite (i.e., even for the lowest employed concentration −10% of SYL244FP). In other words, some fraction of ARP remains amorphous after the recrystallization process. The dielectric signature of uncompleted recrystallization of ARP is the presence of the residual α′-relaxation process in the dielectric loss spectra (see red spectra in Figure 6a,b). The higher the content of SYL244FP, the greater the extent to which the API fraction remains amorphous after recrystallization. To compare the recrystallization tendency of ARP in silica composites, the normalized values of Δε_N_ are plotted as a function of temperature in Figure 6d. The normalization has been performed as follows: Δε_N_ = Δε(*T*)/Δε(*T* = 312 K).

**Figure 7:**
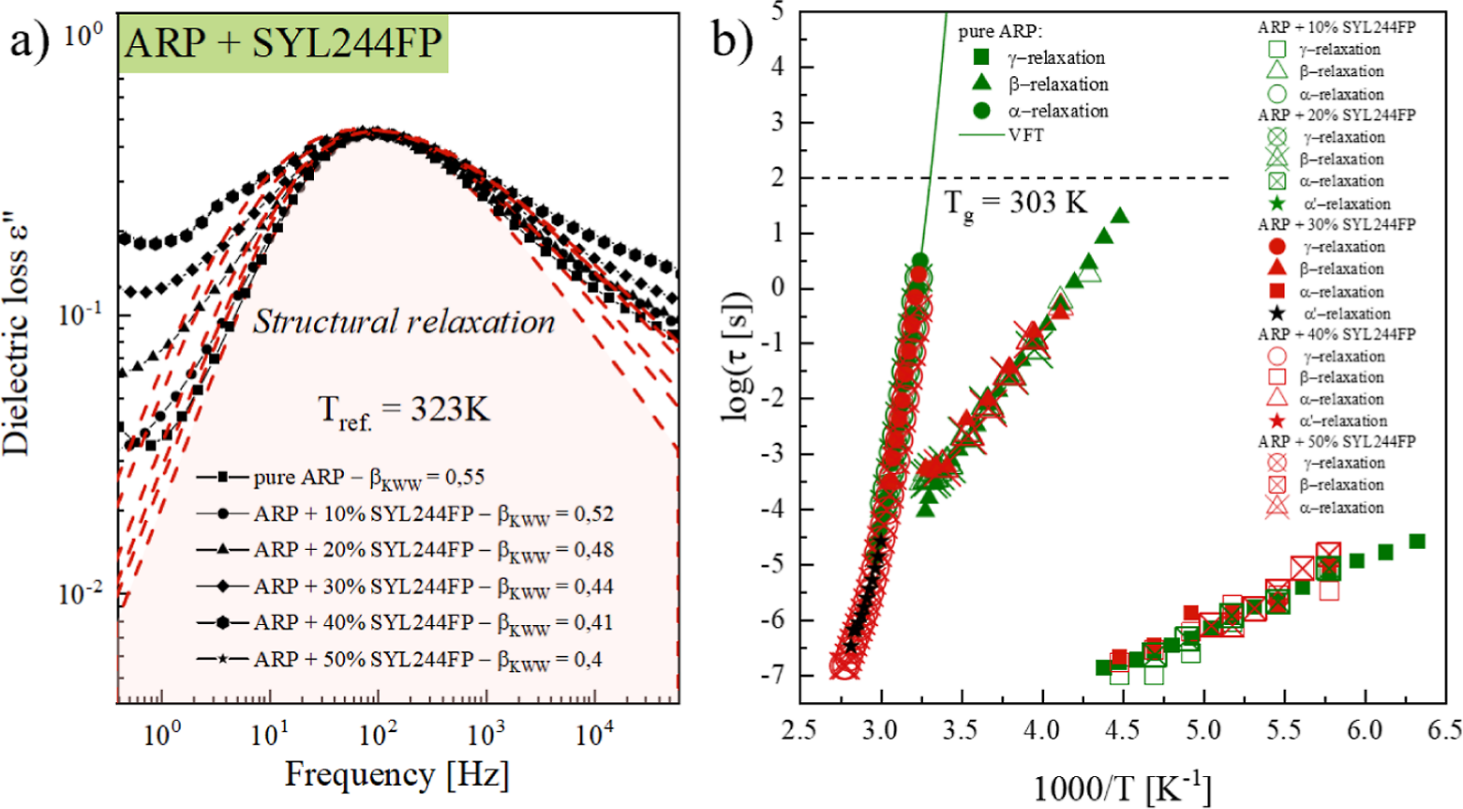
Panel (a) presents a comparison of the dielectric spectra of various concentrations of the ARP + SYL244FP systems recorded at *T* = 313 K. The dashed red lines represent the KWW fit to the α-peak with a value of β_KWW_ given in the legend. Panel b presents the relaxation map of studied systems ARP + SYL244FP. The VFT equation was applied to describe structural relaxation times.

Next, we compared the spectra registered at a reference temperature of 323 K for samples characterized by various silica content. As shown in Figure 7, with increasing the amount of SYL244FP, the α-relaxation peak of ARP becomes broader, which is reflected by a smaller β_KWW_ parameter, i.e., β_KWW_ of pure ARP oscillates around 0.55, and it drops down to 0.4 for a sample containing 50% of SYL244FP. Such behavior is associated with an increase in heterogeneity in the sample.^55,56^

The next step of the dielectric loss spectra analysis is to investigate how silica affects the temperature dependences of the α-, β-, and γ-relaxation times. For this purpose, a similar analysis as that presented in Section 3.2 was performed. The asymmetric α-relaxation as well as the symmetric β- and γ-relaxation processes were fitted using the HN and the CC functions, respectively. The obtained fit parameters were subsequently employed to calculate the τ_α_, τ_β_, and τ_γ_ following eq 2. Determined by this approach, the temperature evolutions of α-, β-, and γ-relaxation times of ARP-SYL244FP composites are plotted in Figure 7b. As noted, the employed silica does not significantly impact the temperature dependencies for both structural or secondary relaxation times of ARP. No significant changes in the molecular dynamics of the tested API in MS composites suggest that the silica modifies the physical stability of ARP by affecting its nucleation. The performed analysis also proved that the α′-relaxation process visible after recrystallization is associated with the structural α-relaxation of the nonrecrystallized fraction of ARP. This conclusion was drawn from the continuation of the temperature evolution of τ_α_ by τ_α’_ visible in Figure 7b.

### 3.5 Effect of Annealing on the Formation of Different Polymorphs of ARP from the Systems Containing SYL244FP

So far, our research has demonstrated that the primary mechanism behind the enhanced physical stability of ARP in MS composites is suppressed nucleation. Therefore, it would be interesting to see whether the nucleation time affects the polymorph characteristics of ARP. For this reason, a series of calorimetric studies have been performed on samples containing 0, 10, 30, and 50% SYL244FP. Each sample was subjected to three types of tests. During these experiments, the most important was the annealing step conducted at a temperature 6 K higher than ARP’s *T*_g_—the temperature at which the maximum nucleation was noted (data not shown).

In all types of experiments, the first step includes heating the sample at a rate of 10 K/min from 298 to 433 K followed by quick (with a rate of 20 K/min) cooling to 298 K. This step aimed to melt and quench the ARP. After that, in the first type of experiment, the sample was reheated to 438 K with 10 K/min (experiment without annealing). In the second and third types of experiments, the reheating step was interrupted by an additional isothermal step at 313 K. The annealing time was set to 4 or 20 h, for the second and third types of experiments, respectively. After this step, the reheating run was continued to 438 K at a 10 K/min rate. The chosen annealing temperature corresponds to the maximum of the ARP’s nucleation curve. Thus, any modification in the melting behavior of the annealed ARP in the composite should indicate the role of MS in ARP nucleation.

**Figure 8:**
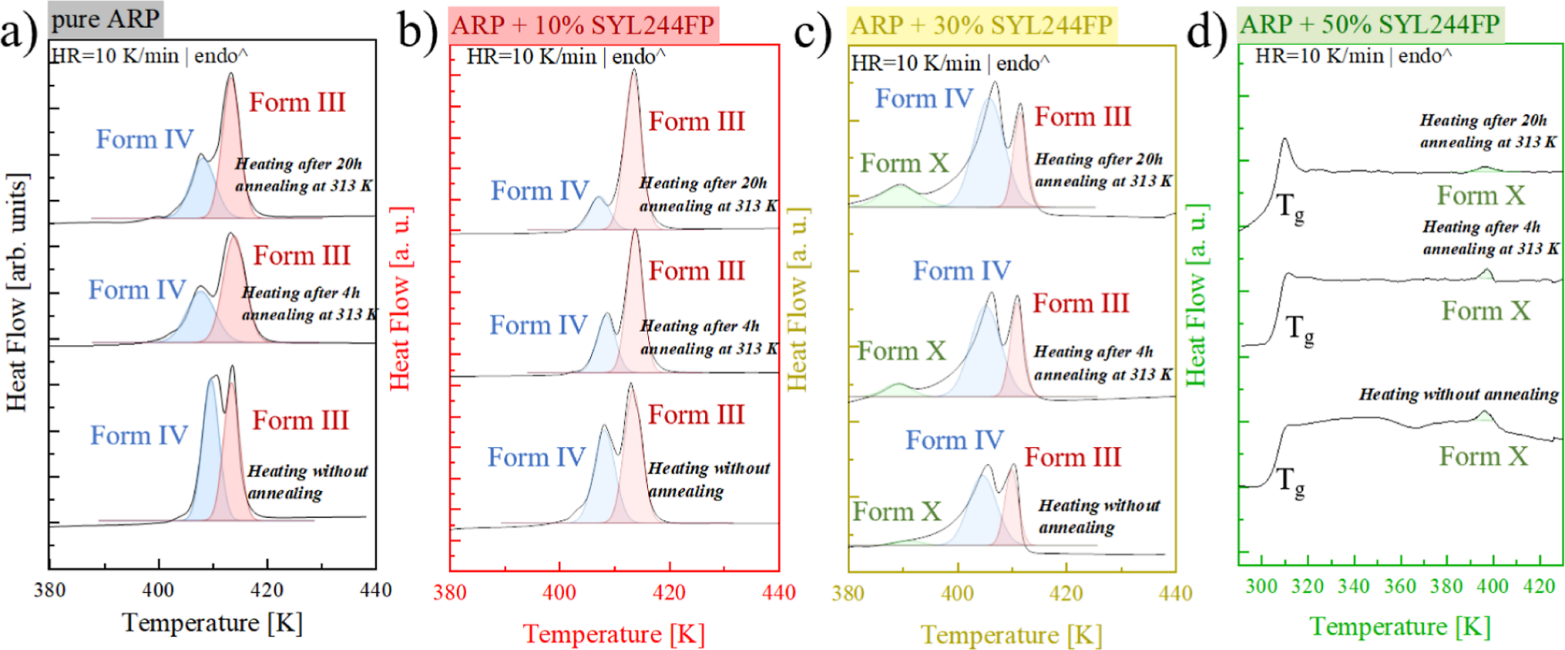
DSC thermograms obtained for quenched samples and after the finished isothermal measurements for (a) pure ARP, (b) ARP + 10% SYL244FP, (c) ARP + 30% SYL244FP and (d) ARP + 50% SYL244FP.

The obtained results are summarized in Figure 8. Panel a of this figure refers to a neat ARP and its behavior during the annealing procedure. The significant increase of fraction III over those of other polymorphic forms can be noted by elongating the annealing time. When the small concentration of MS is considered (i.e., up to 27% that speeds up the recrystallization of the API), one can observe an increase of fraction III over polymorphic form IV. On the other hand, for a composite containing 30 wt % of SYL244FP, a drastic change in the melting behavior of the recrystallized sample is observed. Now, form IV dominates; however, some substantial contribution of other, so far undefined polymorph, appears. Furthermore, the annealing brings more nuclei of IV and undefined polymorphs. It is reflected in the more pronounced melting peaks at 385 and 403 K compared to the nearly unchanged melting of form III. A further increase in the MS loading leads to the vanishing of the III ARP polymorphic form. Instead, a slight crystallization to some other polymorph takes place. This is visualized in Figure 8d, where the DSC thermograms of ARP + 50 wt % of SYL244FP are summarized.

**Figure 9:**
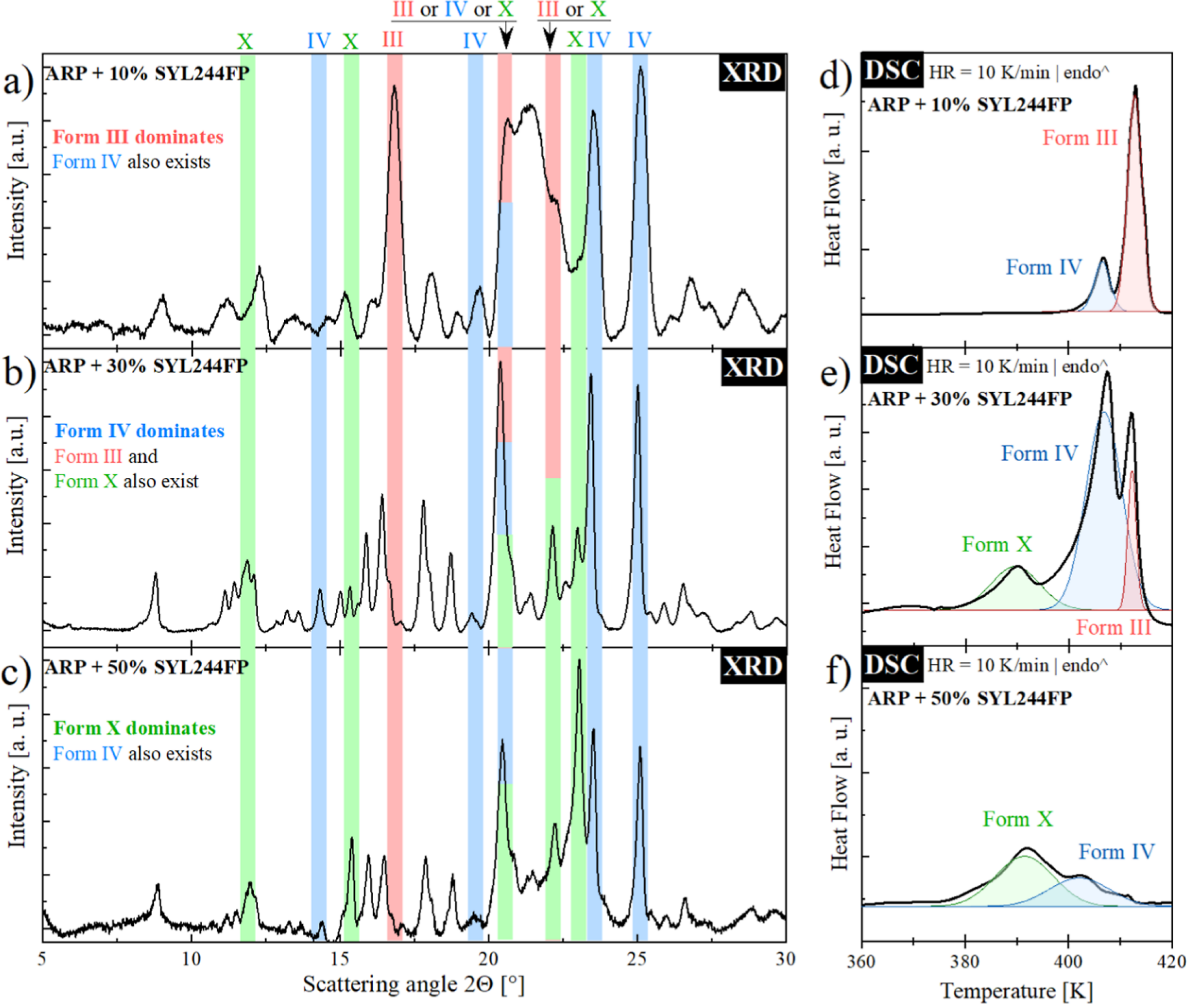
(a–c) XRD patterns and (d–f) DSC thermograms of ARP + MS composites.

Herein, it would be interesting to identify the ARP polymorph existing in compositions containing 30 and 50% wt MS. For this purpose, the XRD measurements have been performed. As shown in Figure 9, recrystallization of ARP from 10 wt % composition indeed brings III and IV polymorphs, while the significant contribution of the former one is observed. However, when the MS loading is increased to 30 wt %, three polymorphs exist in the sample: III, IV, and form X, discovered for the first time in ref (26). Further increase in MS concentration results in crystallization to form X. At the same time, XRD signals from form IV are also observed, while the formation of III polymorph is entirely suppressed.

The results presented in this section indicate that the visible modifications in the ARP’s physical stability after the employment of MS are associated with the modifications in the API nucleation.

## 4 Conclusions

This paper investigated the impact of commercially available MS material, SYL244FP—on the physical stability of supercooled ARP. Our studies revealed unusual recrystallization of API for various content of MS. It has been shown that depending on the concentration of SYL244FP, this additive can trigger, delay, or even block the API recrystallization. Low silica content accelerates the recrystallization of ARP. Using the intermediate content of the MS (i.e., between 27 and 65 wt %), one can inhibit the recrystallization of API. At the same time, high silica concentrations (i.e., >65 wt %) guarantee high physical stability of the drug. A series of calorimetric and dielectric studies were performed to determine the molecular origin of these results. Dielectric and calorimetric experiments indicated that the examined silica material (in any measured concentration) does not modify either the glass transition temperature of ARP or the temperature evolution of its structural (α) or secondary (β and γ) relaxation times. Consequently, the effect of additives on the molecular dynamics of ARP has been excluded from the factors governing the physical stability of ARP. Instead, two other molecular sources of the observed effects can be considered. One is the “immobilization” of the drug molecules on the surface of the silica. The visible effect of this mechanism is an increasing fraction of amorphous API after recrystallization from composites of higher MS content. On the other hand, it has been shown that the silica material affects ARP nucleation. At low concentrations, the MS supports forming the III polymorphic form of ARP. However, when the amount of SYL244FP is ≥ 27 wt %, the formation of the III polymorphic form is no longer favorable. This is because the nuclei of forms IV and X are preferred. Moreover, the crystal growth of forms IV and X takes longer in comparison to that of form III. Additionally, a significant modification in the recrystallization tendency of amorphous ARP was observed when various silica contents were employed. The presented finding demonstrates that by changing the MS loading and controlling the experiment conditions, one can tune the physical state of the ARP.
